# Deciphering psychobiotics’ mechanism of action: bacterial extracellular vesicles in the spotlight

**DOI:** 10.3389/fmicb.2023.1211447

**Published:** 2023-06-15

**Authors:** Layla Bleibel, Szymon Dziomba, Krzysztof Franciszek Waleron, Edward Kowalczyk, Michał Seweryn Karbownik

**Affiliations:** ^1^Department of Pharmacology and Toxicology, Medical University of Lodz, Łódź, Poland; ^2^Department of Toxicology, Medical University of Gdansk, Gdańsk, Poland; ^3^Department of Pharmaceutical Microbiology, Medical University of Gdansk, Gdańsk, Poland

**Keywords:** probiotics, psychobiotics microorganisms, postbiotic, neuropsychiatric disorder, mental health, extracellular vesicles, gut brain axis, mechanism of action

## Abstract

The intake of psychobiotic bacteria appears to be a promising adjunct to neuropsychiatric treatment, and their consumption may even be beneficial for healthy people in terms of mental functioning. The psychobiotics’ mechanism of action is largely outlined by the gut-brain axis; however, it is not fully understood. Based on very recent studies, we provide compelling evidence to suggest a novel understanding of this mechanism: bacterial extracellular vesicles appear to mediate many known effects that psychobiotic bacteria exert on the brain. In this mini-review paper, we characterize the extracellular vesicles derived from psychobiotic bacteria to demonstrate that they can be absorbed from the gastrointestinal tract, penetrate to the brain, and carry the intracellular content to exert beneficial multidirectional action. Specifically, by regulating epigenetic factors, extracellular vesicles from psychobiotics appear to enhance expression of neurotrophic molecules, improve serotonergic neurotransmission, and likely supply astrocytes with glycolytic enzymes to favor neuroprotective mechanisms. As a result, some data suggest an antidepressant action of extracellular vesicles that originate even from taxonomically remote psychobiotic bacteria. As such, these extracellular vesicles may be regarded as postbiotics of potentially therapeutic application. The mini-review is enriched with illustrations to better introduce the complex nature of brain signaling mediated by bacterial extracellular vesicles and indicates knowledge gaps that require scientific exploration before further progress is made. In conclusion, bacterial extracellular vesicles appear to represent the missing piece of the puzzle in the mechanism of action of psychobiotics.

## 1. Introduction

During the last few decades, studies have revealed the fascinating connection between human gut microorganisms and neuropsychological functioning. The gut is home to a unique and diverse community of microbes, collectively known as the gut microbiota. This composed microecosystem plays a crucial role in our overall condition, including mental health (Cuesta et al., 2021). Probiotics—being defined as “live microorganisms which when administered in adequate amounts confer a health benefit on the host” (Hill et al., 2014)—appear to be effective in advantageous modulation of the gut microbiota composition or function (Hemarajata and Versalovic, 2013; Morelli and Patrone, 2015; Oliphant and Claud, 2022). More specifically, the term psychobiotics (Dinan et al., 2013) has been coined to differentiate the gut microbiota modulating preparations that influence the brain function and mental health. Although currently psychobiotics encompass both probiotics and prebiotics (nutritional factors for the intestinal microbiota; Sarkar et al., 2016), in this review article, we refer to the original definition of psychobiotics as mental health-benefiting live microorganisms (Sarkar et al., 2016), such as *Lactobacillus helveticus* and *Bifidobacterium longum* (Messaoudi et al., 2011), or *Akkermansia muciniphila* (Ding et al., 2021), among others.

Recent research shed light on the role of psychobiotics on the gut microbiome and the diverse range of neuropsychiatric disorders. Individuals suffering from depression and anxiety tend to have lower abundance of beneficial gut bacteria with their functional impairment (Jiang et al., 2015; Aizawa et al., 2016; Kelly et al., 2016; Peter et al., 2018; Kazemi et al., 2019), while taking probiotics has been found to reduce depressive and anxiety symptoms (Huang et al., 2017; Ng et al., 2018; Reis et al., 2018; Goh et al., 2019; Liu et al., 2019; Chao et al., 2020; Cohen Kadosh et al., 2021; El Dib et al., 2021). Also, healthy people may benefit from the use of psychobiotics in terms of mental functioning (Messaoudi et al., 2011; Huang et al., 2017; Chao et al., 2020). Moreover, psychobiotics appear to reverse the neurocognitive deterioration in Alzheimer’s disease and mild cognitive impairment (Den et al., 2020; Liu et al., 2023). These findings provide compelling evidence that bacterial psychobiotics may play a vital role in neuropsychiatric treatment and general well-being.

The gut-brain axis (GBA) has been outlined as a framework for mechanism of action of psychobiotics. GBA is a bidirectional communication network between the intestine (together with the residing microbiota) and the brain. This communication is mediated by multiple pathways and messengers, such as the vagus nerve (Breit et al., 2018), the hypothalamic–pituitary–adrenal axis (Frankiensztajn et al., 2020), microbiota-derived neurotransmitters and their precursors (Tillmann et al., 2018; Chen et al., 2021), neurotrophic factors (Ait-Belgnaoui et al., 2014; Mohammadi et al., 2019; Heidarzadeh-Rad et al., 2020), specific bacterial metabolites such as short chain fatty acids (O’Riordan et al., 2022; Karbownik et al., 2023), and immune system, including the modulation of cytokine release (Mohammadi et al., 2019; Rutsch et al., 2020; Partrick et al., 2021). However, the psychobiotics’ mechanism of action is still a mystery to the scientists. Recently, another factor has emerged as potentially shaping the GBA function following the use of psychiobiotics. This factor includes bacterial extracellular vesicles (EVs). It appears that EVs represent the missing piece of the puzzle in GBA and the mechanism of action of psychobiotics.

Psychobiotic bacteria within the gut—as all the living cells—produce and release nanosized EVs carrying bacterial cellular components (Cuesta et al., 2021). Bacterial EVs contribute to communication not only between the microbes, but also at the inter-kingdom level to affect the host cells (Haas-Neill and Forsythe, 2020; Cuesta et al., 2021; Pirolli et al., 2021). Bacterial EVs are small enough to be absorbed from the gastrointestinal tract and penetrate to the brain. In this way, EVs cargo a range of foreign bioactive compounds to affect the central nervous system (CNS) function. In this review, we decipher the role of bacterial EVs in the mechanism of action of psychobiotics by characterizing the nature of probiotic EVs, suggesting the way of their distribution to the CNS, and providing evidence for their action therein. We argue that bacterial EVs represent the novel mechanism of action of psychobiotics and highlight the future directions in relevant research.

## 2. Biogenesis and characteristics of bacteria-derived EVs

**Figure 1:**
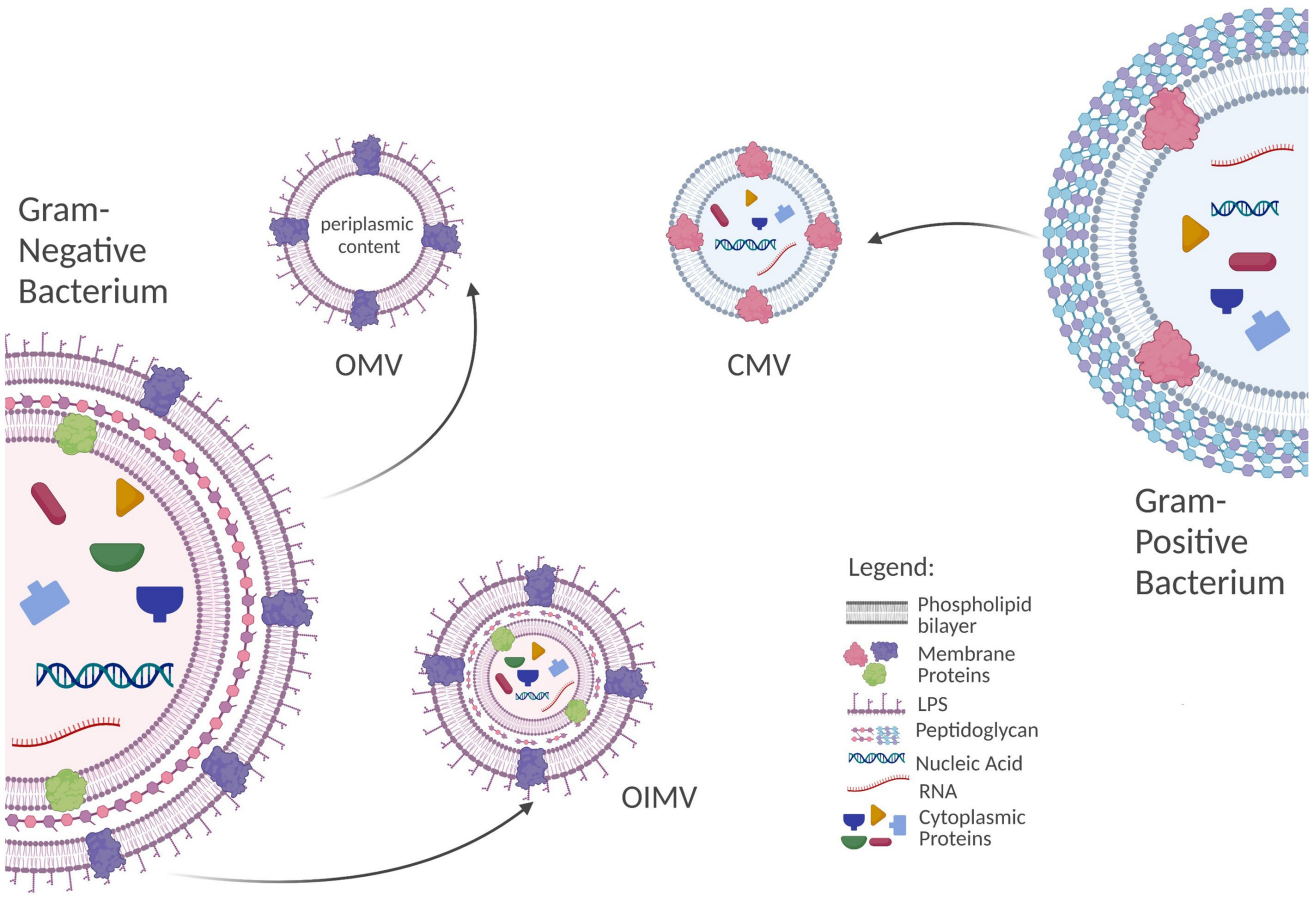
Biogenesis and characteristics of bacteria-derived extracellular vesicles (EVs). Outer-inner membrane vesicles (OIMVs) are formed from Gram-negative bacteria through blebbing or in “explosive cell lysis” process. OIMVs are considered to contain all functional components of parental cells including lipopolysaccharide (LPS) and the fragments of chromosomal deoxyribonucleic acid. Outer membrane vesicles (OMVs) are the most abundant vesicle type secreted by Gram-negative bacteria. OMVs are formed through blebbing and predominantly contain periplasmic content of the parental cell. In the case of Gram-positive bacteria, the mechanism of cytoplasmic membrane vesicles (CMVs) generation is based on selective degradation of peptidoglycan cell wall layer. It might lead to the cell death which is termed “bubbling cell lysis” Created with BioRender.com.

Bacteria have been reported to secrete several types of vesicles. While their biogenesis has not been completely explained, the structure and cargo of the bacterial EVs is dependent on the parental cell and is likely reflected in pharmacological effect under exposition (Toyofuku et al., 2019). In this section, we present only the outline of the current state of knowledge on this topic (Figure 1).

Outer membrane vesicles (OMVs) are the main type of EVs released by Gram-negative bacteria. OMVs are formed from the outer membrane of the cell and are packed with periplasmic material. Some works have also reported the presence of cytosolic proteins as well as genetic material [deoxyribonucleic (DNA) and ribonucleic acids (RNA)] in OMVs isolates (Schwechheimer and Kuehn, 2015; Lee et al., 2016). The secretion of OMVs might be initiated by the local polarization of the membrane (Mashburn-Warren et al., 2008) or intercalation of chemicals into outer membrane (Kobayashi et al., 2000; Florez et al., 2017). Both triggers are assumed to disturb the curvature of membrane which leads to OMVs formation. Increased turgor pressure in periplasmic space, due to the accumulation of biochemicals like misfolded proteins or phospholipids, have also been associated with outer membrane vesiculation (McBroom and Kuehn, 2007; Roier et al., 2016). Small quorum-sensing molecules and proteins additionally stimulate vesicle production (McMillan and Kuehn, 2021).

The OIMVs are double bilayered vesicles, enveloping a fragment of cytosol with a coat composed of inner (cellular) and outer membrane. Thus, OIMVs are considered to contain all functional components of parental cell including lipopolysaccharide (LPS) and the fragments of chromosomal DNA (Baeza et al., 2021). OIMVs might be formed similarly to OMVs through blebbing as a result of autolysin activity that transiently breaks down peptidoglycan to allow OIMV release (Pérez-Cruz et al., 2013; McMillan and Kuehn, 2021). Alternatively, OIMVs might be secreted in “explosive cell lysis” process (Turnbull et al., 2016; Baeza et al., 2021). OIMVs are less abundant fraction than OMVs (their content does not typically exceed few percent of total EVs population during the logarithmic growth phase; Pérez-Cruz et al., 2013; Baeza et al., 2021). Although the size of both types of vesicles is dependent on bacterial species and strain, the diameter of OIMVs typically exceeds 100 nm, and OMVs are usually smaller than OIMVs (Pérez-Cruz et al., 2013).

In the case of Gram-positive bacteria, the mechanism of cytoplasmic membrane vesicles (CMVs) generation is based on selective degradation of thick peptidoglycan cell wall layer. It might lead to the death of the cell which is termed “bubbling cell lysis” (Liu et al., 2018; McMillan and Kuehn, 2021). It is assumed to be analogous to explosive cell lysis occurring in Gram-negative bacteria. Both are initiated by phages or stress conditions like DNA damage (SOS response) or perforation of peptidoglycan by exogenous factors (e.g., enzymes or antibiotics). In response, the expression of endolysins and fragmentation of peptidoglycan occurs. Perforation of thick peptidoglycan in Gram-positive bacteria leads to the “leakage” of cell interior (through the pores) in a form of vesicles (“bubbles”) while in Gram-negative the process is more rapid (cell “explodes”). Nevertheless, CMVs released in this process typically feature 20–200 nm in size and contain all cellular components of parental cell, and reflects the cell condition at the time of death (Toyofuku et al., 2019).

## 3. Signaling and distribution of psychobiotic EVs to the brain

**Figure 2:**
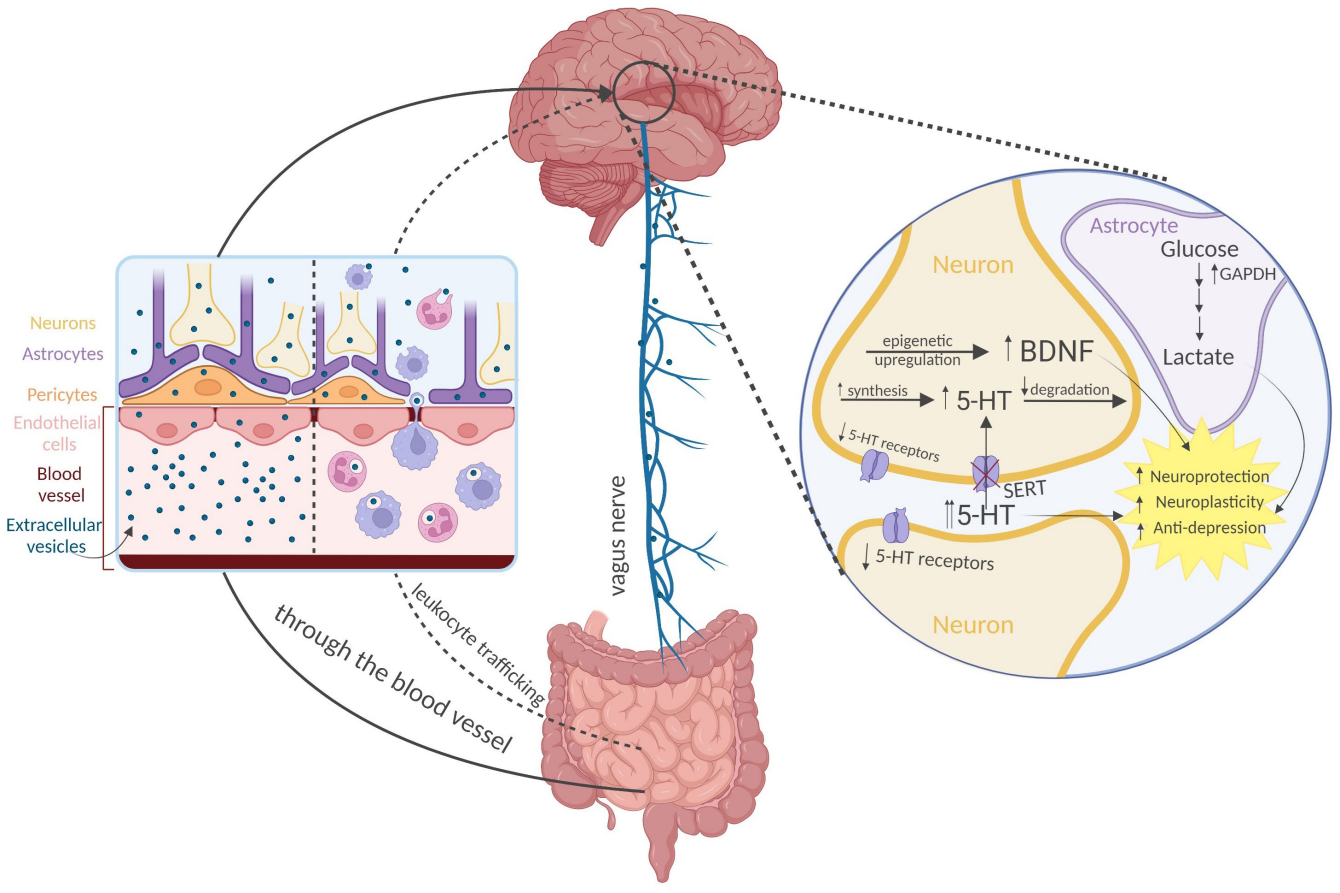
Distribution and action of the psychobiotic bacteria-derived extracellular vesicles (EVs) on the brain. Direct crossing of the blood–brain barrier appears to be the predominant way of EVs transport to the brain. Other routes include vagal nerve transport and activated leukocyte trafficking. According to the current evidence, EVs from psychobiotic bacteria produce antidepressant-like effect mediated by epigenetic upregulation of neurotrophic factors (e.g., brain-derived neurotrophic factor, BDNF), modulation of serotonergic system expression, and possibly by supplementation the astrocytes with glycolytic enzymes (glyceraldehyde 3-phosphate dehydrogenase, GAPDH). 5-HT—serotonin, and SERT—5HT transporter Created with BioRender.com.

Extracellular vesicles may represent the efficient nanostructure for transport of bacterial bioactive compounds to the human brain. After being absorbed from the gastrointestinal tract (Stentz et al., 2018), the most likely mechanism of EVs distribution to the CNS is by (1) crossing the blood–brain barrier (BBB; Matsumoto et al., 2017). Other potential ways include (2) vagal nerve transport and (3) activated leukocyte trafficking to the brain (Figure 2).

A set of *in vitro* experiments have been performed to document the EVs transport through the intact BBB. In the study by Morad et al., an *in vitro* static BBB model has been constructed. This was a two-channel microfluidic culture device that contained a vascular channel lined by induced pluripotent stem-derived human microvascular endothelial cells, which were separated by a porous extracellular matrix-coated membrane from an abluminal channel containing primary human astrocytes and pericytes (Morad et al., 2019). In this way, the model could replicate the barrier function of *in vivo* intact human BBB (Park et al., 2019). By labeling tumor-derived 150 nm size EVs and flowing them through the vascular channel, a fluorescent signal was then detected in the abluminal chamber and increased significantly over time. Fluorescence microscopy analysis confirmed the presence of EVs that were taken up by astrocytes in the abluminal chamber. These findings demonstrate that EVs can interact with endothelial cells under flow conditions and continuously cross the endothelial layer through transcytosis (Morad et al., 2019).

Furthermore, a zebrafish model was used for *in vivo* studies to explore transcytosis of EVs through the BBB. Zebrafish develop a mature BBB at 3 days postfertilization and are a suitable model for BBB studies. Intracardiac injection of the brain-seeking EVs into zebrafish was performed and their distribution in the brain was monitored through live imaging. The results of this study showed that EVs were taken up by multiple cells within the brain parenchyma, proving their ability to cross the BBB *in vivo*. Furthermore, transport of EV-containing endocytic vesicles within endothelial cells could be observed using time-lapse imaging. As in transcytosis, these vesicles moved toward the plasma membrane and fused with the membrane. Most importantly, the BBB remained intact throughout the duration of these experiments, highlighting the absence of an inflammatory process (Morad et al., 2019).

Although several other papers similarly documented the passage of EVs of various origin through the intact BBB (Banks et al., 2020; Jakubec et al., 2020; Morales-Prieto et al., 2022; Zhou et al., 2022), the other mechanisms appear to exist as well. A second pathway that bacterial EVs may penetrate to the brain is through the vagal nerve. *Paenalcaligenes hominis* isolated from the feces of elderly people and aged mice were transplanted into young mice, and bacterial EVs-mediated cognitive impairment and colonic inflammation was noted (Lee et al., 2020). Specifically, Lee et al. (2020) orally gavaged conjugated *P. hominis* EVs or LPS in mice. The results showed that conjugated EVs and LPS were detectable in microglial cells and in the pyramidal region of the hippocampus. Furthermore, conjugated EVs were more abundant than LPS. However, following vagotomy, conjugated EVs abundance in this region was significantly reduced, unlike the conjugated LPS, which was not affected. Additionally, bacterial 16S ribosomal DNA levels in the hippocampus were increased after oral gavage of *P. hominis* or its EVs, whereas the process was inhibited by vagotomy. These findings suggest that EVs of *P. hominis* are orally absorbed and may be able to exert their actions on the brain at least partially through transport via the vagus nerve (Lee et al., 2020).

Other putative mechanisms that have not been extensively studied include EVs crossing the BBB via activated leukocytes trafficking to the brain. Bacterial peptidoglycans, which are a component of EVs, have been identified in the brains of patients with multiple sclerosis, which are heavily infiltrated with blood derived leukocytes. This suggests that these immune cells could be a source of EVs entry into the CNS, although it was not directly investigated (Schrijver et al., 2001). This mechanism of action is also common for viruses’ (size 20–200 nm) ability to reach and infect the brain (Wurdinger et al., 2012; Branton et al., 2013).

## 4. Effects of EVs derived from psychobiotics on the CNS

Mounting evidence confirms that pathogenic bacteria EVs produce deleterious effects on the CNS function, whereas probiotic bacteria EVs exert beneficial effects on peripheral tissues (Cuesta et al., 2021; Pirolli et al., 2021). This provides indirect evidence for advantageous CNS action of EVs from psychobiotics. Depending on the species of origin, bacterial EVs may have both beneficial and detrimental effects on the CNS, with the latter being more extensively studied (Cuesta et al., 2021). Post-mortem brain samples from patients with Alzheimer’s disease have shown the presence of bacterial nucleic acids that could have been transported in EVs (Emery et al., 2017; Cuesta et al., 2021; Vandendriessche et al., 2021). EVs from the periodontal pathogen *Aggregatibacter actinomycetemcomitans* were shown to cross the BBB after intracardiac injection to mice, carrying bacterial extracellular nucleic acids, which increased expression of tumor necrosis factor alpha, a known pro-inflammatory cytokine, in the brain cortex (Han et al., 2019). On the other hand, probiotic bacteria EVs from lactobacilli (Al-Nedawi et al., 2015; Behzadi et al., 2017; Li et al., 2017; Seo et al., 2018; Choi et al., 2020; Kim et al., 2020), bifidobacteria (Kim et al., 2016), as well as other probiotic genera (de Rodovalho et al., 2020), were found to exert beneficial anti-inflammatory action through various mechanisms as tested in human peripheral cells or animal models relevant to non-CNS disorders.

However, very recent literature provides direct evidence for CNS-relevant advantageous action of EVs from psychobiotics (Figure 2). Choi et al. (2019) investigated the therapeutic potential of EVs derived from *Lactiplantibacillus plantarum* on depression model. The authors induced depressive-like behavior in stressed mice and administered intraperitoneal injections of *L. plantarum* EVs. The results showed a reduction in depressive-like behavior, an increase in brain-derived neurotrophic factor (BDNF) gene expression in the hippocampi, and an additional increase in BDNF expression in a glucocorticoid-suppressed mouse neuronal cell line culture following *L. plantarum*-derived EVs treatment. This evidence was the first to suggest that EVs from psychobiotics may be beneficial at both molecular and functional levels. This indicated the potential for psychobiotic EVs as a therapeutic option for depression, and supported the notion that EVs play a significant role in the psychobiotics’ mechanism of action (Choi et al., 2019).

A follow-up study conducted by the same research team has suggested that the effect is not limited to EVs from just one bacterial psychobiotic strain. Choi et al. (2022) induced chronic stress-like conditions in mice and administered EVs isolated from taxonomically different potential psychobiotic bacteria *L. plantarum*, *Bacillus subtilis*, or *Akkermansia muciniphila*. The authors noted that all the EVs produced an anti-depressive-like effect and restored diminished hippocampal BDNF as well as neurotrophin-3 and -4/5 expression; however, the behavioral effect was the most pronounced in case of EVs from *L. plantarum*, and EVs from *A. muciniphila* affected partially different epigenetic regulators of the expression of tested neurotrophic factors than that of *L. plantarum* and *B. subtilis*. The superior effect of *L. plantarum* EVs was not surprising as the authors found particularly remarkable stress-induced decrease in lactobacilli abundance in the gut microbiota in the tested animal model, which produced a gap for therapeutic response from the relevant EVs (Choi et al., 2022).

It becomes clearer that EVs from psychobiotic bacteria produce more versatile CNS-relevant effects than that mediated by neurotrophic factors. A study by Yaghoubfar et al. (2020) has shed light on the effects of EVs from potential psychobiotic *A. muciniphila* (Ding et al., 2021) on expression of serotonergic system in mice hippocampi. Oral treatment with the EVs led to an increase in the messenger RNA expression of the gene encoding rate limiting serotonin (5-HT) synthesizing enzyme tryptophan hydroxylase, whereas a decrease in degrading enzyme monoamine oxidase was noted. Also, 5-HT level was found to be increased, possibly extracellularly, as the expression of 5-HT transporter reduced. At the same time, the expression of some 5-HT receptors was decreased. As the treatment with *Akkermansia* EVs in the experiment lasted for 4 weeks (Yaghoubfar et al., 2020), the resulted serotonergic regulation may be comparable to that following chronic and therapeutically relevant treatment with antidepressants (Artigas, 2013; Gray et al., 2013; Bowman and Daws, 2019).

Another proposed mechanism for the beneficial effects of EVs from psychobiotics involves glycolytic enzymes. As a byproduct of glycolysis, astrocytes produce lactate, and it happens regardless of sufficient oxygen availability (Takahashi, 2021). Astrocyte-derived lactate is believed to fuel neurons, support synaptic plasticity processes (Suzuki et al., 2011) and its transport may prevent depression (Yao et al., 2023). Thus, high glycolytic activity in astrocytes may be beneficial (Takahashi, 2021). In a study by Bajic et al. (2020), EVs released by the dairy isolate *L. plantarum* were found to be enriched in enzymes involved in central metabolic pathways, including glycolysis, in particular, glyceraldehyde 3-phosphate dehydrogenase. This suggests that glycolytic enzymes may be supplemented by psychobiotic EVs to astrocytes, thus contributing to neuroprotection, however, a direct causation needs to be examined.

A study by Yang et al. (2022) explored the potential of EVs derived from *L. plantarum* against ischemic brain injury. Although this condition represents an acute clinical event, post-stroke complications typically include depression and cognitive deterioration (Wijeratne and Sales, 2021). The results showed that EVs from *Lactiplantibacillus* significantly reduced brain damage and improved neurological function in mice following a stroke. Moreover, they reduced infarct size and decreased neurological deficits. The mechanism of this protective effect involved the regulation of a specific microRNA, miR-101a-3p, and its downstream targets, c-Fos and transforming growth factor-β, leading to the inhibition of neuron apoptosis. Importantly, miR-101a-3p could also serve as a marker for neurological recovery in ischemic stroke patients. This finding may further shed light on the EVs-mediated mechanism of CNS-relevant action of psychobiotic bacteria (Yang et al., 2022).

## 5. Discussion

Gut-brain axis outlines complex mechanisms in which psychobiotics exert action on the brain. Until recently, it has not been appreciated that the GBA-mediated action may be additionally evoked through bacterial secreted EVs; this topic has been neglected in majority of recent review papers in the area of psychobiotics’ mechanism of action (Del Toro-Barbosa et al., 2020; Dey and Mookherjee, 2021; Johnson et al., 2021; Tremblay et al., 2021; Yang et al., 2021; Magalhães-Guedes, 2022; Suda and Matsuda, 2022). Nevertheless, here we provide substantial evidence supporting such supposition. EVs from psychobiotic bacteria are small enough to be absorbed from the gastrointestinal tract and transported to the brain, where they can interact with the CNS components, affecting various range of brain processes. EVs from psychobiotics—even originated from taxonomically remote bacteria—may produce antidepressant effects. This may be through modulation of the expression of neurotrophic factors (Choi et al., 2019, 2022), neurotransmitter regulation (Yaghoubfar et al., 2020), or possible supplementation of the astrocytes with glycolytic enzymes (Bajic et al., 2020), among the investigated mechanisms. There is also a large body of evidence supporting anti-inflammatory action of probiotic EVs (Al-Nedawi et al., 2015; Kim et al., 2016, 2020; Behzadi et al., 2017; Li et al., 2017; Seo et al., 2018; Choi et al., 2020; de Rodovalho et al., 2020), however, none was performed in the CNS-relevant model. Collectively, EVs appear to intermediate a great deal of known mechanisms within the GBA. Moreover, psychobiotic bacteria-derived EVs may represent the “concentrated” messenger to the brain, as some of their effects were multiplied in comparison to administration of parental psychobiotic bacteria (Yaghoubfar et al., 2020). In this light, EVs may represent the missing piece of the puzzle in mechanism of action of psychobiotics.

Psychobiotics encompass Gram-positive and Gram-negative bacteria. Despite their differences, numerous bacteria from both these groups were shown to secrete EVs and to feature similar pharmacological effect in the GBA (Choi et al., 2022). On the other hand, EVs even from the same bacteria species (e.g., *Escherichia coli*) may have the opposite strain-dependent pro- (Imamiya et al., 2023) or anti-inflammatory properties (Güttsches et al., 2012), and this phenomenon results from the altered form of LPS expressed by the parental bacteria (Güttsches et al., 2012). Little is known about specific ingredients and markers of bacterial EVs [e.g., fatty (Schroeder and Bäckhed, 2016; Champagne-Jorgensen et al., 2021) or ribonucleic acids (Lee et al., 2020), kinases (Gradowski et al., 2020), and other enzymes (Bajic et al., 2020)] that translate to the properties of psychobiotics. Scientific efforts toward their identification are urgently needed to understand EVs-mediated mechanism of action.

Limited knowledge on the biogenesis of bacterial EVs and their still evolving classification make it difficult to unequivocally identify the type of vesicles responsible for certain CNS-relevant effects. Since 2018, the updated recommendation of International Society of Extracellular Vesicles considering, *inter alia*, isolates characterization has increased the reporting standards (Théry et al., 2018), with the hope for deciphering relationship between probiotic EVs quality and their pharmacological effects. Still, the employment of analytical techniques enabling subpopulations differentiation is highly desirable (Steć et al., 2022). In addition, the quantity of bacterial EVs that could represent their CNS-effective but safe dose requires elucidation.

Numerous works have proved intensive secretion of EVs by bacteria under unfavorable stress conditions, e.g., misfolded proteins accumulation (McBroom and Kuehn, 2007; Roier et al., 2016), DNA damage or perforation of peptidoglycan, and reflecting cell condition at the death (Toyofuku et al., 2019). It is intriguing how such stress-induced EVs produced by psychobiotics may exert any beneficial effect to the human. The phenomenon may be partially explained by the nature of parental cells (Güttsches et al., 2012; Ashrafian et al., 2019). However, the bacterial EVs may be produced also under physiological conditions during their logarithmic growth (Baeza et al., 2021; Laurin et al., 2022). Moreover, the reason for bacteria to produce EVs remains a major paradox (McMillan and Kuehn, 2021), and these issues require further studies.

Also, the EVs from psychobiotic bacteria tested in the CNS-relevant models have been obtained in precisely defined conditions (Choi et al., 2019; Yaghoubfar et al., 2020; Choi et al., 2022). Nevertheless, EVs composition and size can change drastically, depending on environment and growth conditions (Ñahui Palomino et al., 2021). The potential effect of variable host physiological status on probiotic bacteria EVs generation and action requires further attention. Moreover, psychobiotic bacteria EVs may change the properties of other bacterial vesicles (McMillan and Kuehn, 2021) or host-derived EVs (Imamiya et al., 2023), further entangling their mechanism of action.

Although convincing evidence supports the role of bacterial EVs in the psychobiotics’ mechanism of action, many questions need to be addressed, with some being raised above. Once solved, psychobiotic bacteria-derived EVs have potential to become a new generation postbiotics.

## Author contributions

MK: conceptualization, supervision, and project administration. LB and MK: methodology and visualization. LB, SD, KW, and MK: literature search and writing—original draft. EK: funding acquisition. LB, SD, KW, EK, and MK: writing—review and editing. All authors contributed to the article and approved the submitted version.

## Funding

The research was supported by Medical University of Lodz (Łódź, Poland; grant number 503/5-108-03/503-51-001-19-00). The funder had no role in the design of the study; in the collection, analyses, or interpretation of data; in the writing of the manuscript; or in the decision to publish it.

## Conflict of interest

The authors declare that the research was conducted in the absence of any commercial or financial relationships that could be construed as a potential conflict of interest.

## Publisher’s note

All claims expressed in this article are solely those of the authors and do not necessarily represent those of their affiliated organizations, or those of the publisher, the editors and the reviewers. Any product that may be evaluated in this article, or claim that may be made by its manufacturer, is not guaranteed or endorsed by the publisher.
